# Fluoroquinolone resistant bacterial isolates from the urinary tract among patients attending hospitals in Bushenyi District, Uganda

**DOI:** 10.11604/pamj.2020.36.60.18832

**Published:** 2020-06-02

**Authors:** Martin Odoki, Adamu Almustapha Aliero, Julius Tibyangye, Josephat Nyabayo Maniga, Emmanuel Eilu, Ibrahim Ntulume, Eddie Wampande, Charles Drago Kato, Ezera Agwu, Joel Bazira

**Affiliations:** 1 Department of Microbiology and Immunology, Faculty of Biomedical Sciences, Kampala International University-Western Campus, Bushenyi, Uganda,; 2 Department of Microbiology, Faculty of Life Sciences, Kebbi State University of Science and Technology Aliero, Kebbi State, Nigeria,; 3 Department of Microbiology and Immunology, College of Health, Medicine and Life Sciences, King Ceasor University, Kampala, Uganda,; 4 Department of Biochemistry, Faculty of Biomedical Sciences, Kampala International University-Western Campus, Bushenyi, Uganda,; 5 Department of Immunology and Molecular Biology, College of Health Sciences, Makerere University, Kampala, Uganda,; 6 School of Bio-security, Biotechnical and Laboratory Sciences, College of Veterinary Medicine, Animal Resources and Bio-security, Makerere University, Kampala, Uganda,; 7 Department of Microbiology and Immunology, Division of Biomedical Sciences, Kabale University School of Medicine, Kabale, Uganda,; 8 Department of Microbiology, Faculty of Medicine, Mbarara University of Science and Technology, Mbarara, Uganda

**Keywords:** Fluoroquinolone, resistant bacteria, urinary tract infections, Bushenyi District, Uganda

## Abstract

**Introduction:**

bacterial resistance to fluoroquinolones is on the rise globally, bacteria causing urinary tract infections (UTIs) are no exception to this fact. Judicious use of the current antibiotics by clinicians is therefore deemed necessary to combat development of resistance. This study determined fluoroquinolone resistant profiles, multiple antibiotic resistance indices (MARI), factors associated with fluoroquinolone resistance and their strength among patients attending hospitals in Bushenyi District, Uganda.

**Methods:**

this was a cross-sectional study in which a total of 86 bacterial uropathogens isolated previously by standard microbiological methods were subjected to antibiotic susceptibility testing using Kirby Bauer disk diffusion method. Data for factors suspected to be associated with fluoroquinolone resistant UTI were obtained by use of questionnaires.

**Results:**

the most resisted fluoroquinolone was ofloxacin with 29/83 (34.9%), followed by moxifloxacin 27/83 (32.5%), levofloxacin 24/86 (27.9%) and ciprofloxacin 23/86 (26.7%). The bacterial uropathogens that exhibited the highest frequency of fluoroquinolone resistant strains were *P. mirabilis* with 2/3 (66.7%) and *E. faecalis* with 2/3 (66.7%), followed by *E. coli* 19/36 (52.8%), *S. aureus* 13/27 (48.1%), *K. oxytoca* 2/6 (33.3%), *K. pneumoniae* 2/10 (20.0%) and *P. vulgaris* 0/1 (0.0%). All the bacterial uropathogens tested showed MARI of ≥ 0.2. Hospitalization, history of fluoroquinolones use in the last 12 months and wrong prescription of antibiotics were found to bear statistically significant relationships (p < 0.05) with fluoroquinolone resistant UTI.

**Conclusion:**

antibiotic susceptibility testing of the first generation quinolones such as nalidixic acid in hospitalized patients, patients with history of fluoroquinolones' use in the last 12 months and wrong prescription of antibiotics should be adopted to avoid fluoroquinolone abuse. For empiric treatment of UTIs in Bushenyi District, ciprofloxacin still remains the first line of choice among the fluoroquinolone class of antibiotics.

## Introduction

Urinary tract infections (UTIs) are known to be one of the commonest nosocomial and community-acquired bacterial infections [1]. Globally, about 150 million people per year are diagnosed with UTIs, resulting into more than 6 billion US dollars used for health care [2-4]. The World Health Organization (WHO) surveillance report on antimicrobial resistance in 2014 indicated that, nine bacteria of global concern are the main cause of nosocomial and community-acquired infections [5]. Complicated UTI, such as pyelonephritis is most frequently treated with fluoroquinolones [6], although ciprofloxacin is commonly used in the treatment of uncomplicated UTIs [7]. The rapid increase in the use of fluoroquinolones has led to the emergence of ciprofloxacin resistant bacterial uropathogens globally, for uncomplicated UTIs it ranges from 2% to 69% and goes up to 98% for complicated UTIs [8]. Ciprofloxacin resistance in uncomplicated UTIs in selected European countries showed the following values; 4.8%, 20.3%, 30.8%, 7.3% and 15.3% in France, Germany, Spain, Sweden and UK in 2014 respectively [9].

While in an out-patient population with UTIs in the US, ciprofloxacin resistance of 17.1% was observed [10]. In an outpatients department in the Netherlands, a much lower resistance to ciprofloxacin of ∼10% was reported in 2014 [11]. Although a slightly higher resistance of 12% was reported in a study that included both in-patients and out-patients with complicated UTIs in 2004-09 [12]. Fluoroquinolones are among the most frequently prescribed antibiotics in the empirical treatment of UTIs [13]. There is increasing resistance in recent years especially among Gram negative bacteria due to over-prescription of fluoroquinolones [14]. The rates of bacterial resistance to fluoroquinolones varies world over, ranging from greater than 50% community acquired uncomplicated UTI to extremely higher value of 98% for strains responsible for complicated community-acquired UTI. In nosocomial UTI, bacterial uropathogens are more resistant to fluoroquinolones than community-acquired UTI [8]. Uropathogenic *E. coli* (UPEC) is the chief etiological agent in 20% of all fluoroquinolone resistant nosocomial infections [15].

Currently, the greatest threat to global health is antimicrobial resistance (AMR) as declared by WHO, the use of antibiotics is attributed to this course [16]. It´s unfortunate, that quite a few antibiotic drug candidates are in the pipeline [17] and judicious use of the commonly used antibiotics has to be encouraged in order to slow down the current AMR trend [18]. This will also involve dose modifications to achieve desired drug plasma concentration levels, because low ciprofloxacin levels will definitely open the mutant selection window, leading to rapid selection of resistant subpopulations [19]. The concentration of ciprofloxacin in blood and urine is often influenced by age and renal insufficiency [20, 21]. Also, drugs that are prescribed concomitantly may alter the pharmacokinetics of fluoroquinolones, like bioavailability and excretion. Putting more emphasis on age and renal function or modifications of co-medication may lead to desirable drug levels and reduced selection of fluoroquinolone resistant uropathogens. However, modifications of other factors may also play a role.

In Uganda, a resistance of 40/82 (48.8%) to ciprofloxacin was reported in a study carried out in community acquired uropathogens in Gulu, Northern Uganda [22], 23/57 (40.4%) in a study carried out to determine bacteriuria among adult non-pregnant women attending Mulago hospital assessment centre in Uganda [23] and 6/14 (42.9%) resistance was reported in a study carried out to determine the factors associated with community-acquired urinary tract infections among adults attending assessment centre, Mulago hospital Uganda [24]. In Bushenyi District of Uganda, in a study to determine the prevalence and antibiotic susceptibility pattern of bacterial urinary tract infections among suspected diabetic patients attending clinics, a resistance of 36/103 (35.0%) to ciprofloxacin was reported [25]. Empirical treatment of bacterial infections depends on the selection of the most appropriate antibiotics, depending upon regional susceptibility profile, key indicators in the genomic evolutionary trend and efficacy of the antibiotic commonly prescribed in a specific locality [4]. For those reasons therefore, local epidemiological studies are important in the selection of the most suitable antibiotics for empirical treatment, so as to redeem the development of resistance to commonly used drugs. To date there is no detailed data from Bushenyi District, Uganda that outlines uropathogens´ fluoroquinolones resistance profile. This study was therefore designed to determine the fluoroquinolone resistance profile, MARI, factors associated with fluoroquinolone resistance and their strength to the commonly encountered uropathogens among patients attending hospitals in Bushenyi District, Uganda.

## Methods

**Study design:** this was a cross-sectional health-point survey conducted from October, 2017 to January, 2018 on the bacterial uropathogens isolated previously from the urinary tract among patients that attended Kampala International University-Teaching Hospital (KIU-TH), Ishaka Adventist Hospital and Comboni Hospital Kyamuhunga by Odoki *et al*. [26].

**Study variables:** provider questionnaires were administered to collect information from the study participants as regards sociodemographic data such as: age, gender, residence, marital status, level of education, circumcision and sexual intercourse. Data on the health status were obtained by the clinicians through clinical examinations and medical history of the study participants like: hypertension, genitourinary abnormalities, abortion, previous surgery, recurrent UTI, previous hospital admission, family history of UTI, previous UTI, history of fluoroquinolone use in the last 12 months, indwelling catheter, chronic respiratory disease, wrong prescription and incomplete dose of antibiotics. Data on selected factors suspected to be associated with fluoroquinolone resistant UTI such as pregnancy, diabetes mellitus and HIV were obtained through laboratory investigations [26].

**Antibiotic susceptibility testing:** the antibiotic susceptibility was done at Mbarara University of Science and Technology-Teaching Hospital (MUST-TH) microbiology laboratory. Antibiotic susceptibility was performed on bacterial isolates from midstream urine (MSU) using antibiotic discs, according to clinical and laboratory standards institute (CLSI) [27] on Muller Hinton agar. The prepared media was inoculated with bacterial suspension equivalent to 0.5 MacFarland turbidity. The commercially available antibiotic discs containing the following antibiotics: nalidixic acid (30μg), ciprofloxacin (5μg), ofloxacin (5μg), levofloxacin (5μg), moxifloxacin (5μg) (Himedia, India) was aseptically placed on the surfaces of the sensitivity agar plates with a sterile forceps and allowed to stand for 30 mins. The plates were then incubated for 18-24 hrs at 37^º^C. Zones of inhibition after incubation was observed, noted and interpreted according to CLSI [27]. Isolates showing intermediate antibiotic susceptibility were considered to be resistant. *Escherichia coli* (ATCC 25922) and *Staphylococcus aureus* (ATCC 25923) were used as quality control organisms for the antimicrobial susceptibility testing according to CLSI [27].

**Multiple antibiotic resistance indices (MARI):** calculation of MARI was done by dividing the number of antibiotics that the bacterial uropathogen is resistant to, by the total number of antibiotics to which the bacterial uropathogen was tested against [28].

**Data analysis:** it was done by descriptive statistics and regression using IBM SPSS version 20. Descriptive statistics was used to obtain fluoroquinolone resistant profiles and distribution of fluoroquinolones´ resistance among the bacterial uropathogens. The outcome of fluoroquinolone resistant UTI was dichotomized as presence or absence of the fluoroquinolones´ resistance and tested against factors suspected to be associated with fluoroquinolone resistant UTI to assess for associations. Bivariate analysis was applied and all the variables with a *p* value of 0.2 or less were entered into stepwise forward multiple logistic regression model. Interaction and confounding were assessed and values of p ≤ 0.05 were regarded as statistically significant relationships.

**Ethical approval:** the ethical approval of the study was sought from Mbarara University of Science and Technology (MUST, Institutional Research and Ethics Committee (IREC) on Human Research (No. 01/01-17) and final approval was obtained from Uganda National Council for Science and Technology (UNCST) with UNCST registration number: HS 2232. All research protocols were performed in accordance with the ethical standards of committees on human experimentation laid down in the Helsinki declaration of 1964 revised in 2000 [29].

## Results

**Resistance profiles of the bacterial uropathogens to fluoroquinolones:** when the bacterial uropathogens were subjected to the fluoroquinolones, the following resistant profiles were observed; the most resisted fluoroquinolone was ofloxacin with 29/83 (34.9%), followed by moxifloxacin 27/83 (32.5%), levofloxacin 24/86 (27.9%) and ciprofloxacin 23/86 (26.7%). While the first generation quinolone, nalidixic acid was most resisted with 54/86 (62.8%) as compared to any fluoroquinolone tested. The most resistant Gram negative bacterial uropathogen to fluoroquinolones was *E. coli* with 12/36 (33.3%) ciprofloxacin, 16/36 (44.4%) ofloxacin, 11/36 (30.6%) levofloxacin and 13/36 (36.1%) moxifloxacin resistance (Table 1).

**Table 1:** resistance profiles of the bacterial uropathogens to fluoroquinolones

Uropathogens/Antibiotics	NAL	CIP	OFL	LEV	MOX
*E. coli*	22(61.1)	12(33.3)	16(44.4)	11(30.6)	13(36.1)
*K. pneumonia*	7(70.0)	1(10.0)	1(10.0)	1(10.0)	1(10.0)
*K. oxytoca*	4(66.7)	2(33.3)	0(0.0)	2(33.3)	2(33.3)
*P. mirabilis*	0(0.0)	0(0.0)	0(0.0)	2(66.7)	1(33.3)
*P. vulgaris*	1(100.0)	0(0.0)	0(0.0)	0(0.0)	0(0.0)
*S. aureus*	18(66.7)	7(25.9)	12(44.4)	6(22.2)	10(37.0)
*E. faecalis*	2(66.7)	1(33.3)	-	2(66.7)	-
Total	54(62.8)	23(26.7)	29(34.9)	24(27.9)	27(32.5)

Foot note: NAL=nalidixic acid; CIP=ciprofloxacin; OFL=ofloxacin; LEV=levofloxacin; MOX=moxifloxacin

**Distribution of fluoroquinolones´ resistance among the bacterial uropathogens:** when the bacterial uropathogens were subjected to fluoroquinolones, the bacterial uropathogens that exhibited the highest frequency of fluoroquinolone resistant strains were *P. mirabilis* with 2/3 (66.7%) and *E. faecalis* with 2/3 (66.7%), followed by *E. coli* 19/36 (52.8%), *S. aureus*13/27 (48.1%), *K. oxytoca* 2/6 (33.3%), *K. pneumoniae* 2/10 (20.0%) and *P. vulgaris* 0/1 (0.0%) (Table 2). All the bacterial uropathogens tested showed MARI of ≥0.2 (Table 3).

**Table 2:** distribution of fluoroquinolones´ resistance among the bacterial uropathogens

Uropathogens	FQs resistant strains n (%)	Non-FQs resistant strains n (%)	Total n (%)
*E. coli*	19(52.8)	17(47.2)	36(41.9)
*K. pneumoniae*	2(20.0)	8(80.0)	10(11.6)
*K. oxytoca*	2(33.3)	4(66.7)	6(7.0)
*P. mirabilis*	2(66.7)	1(33.3)	3(3.5)
*P. vulgaris*	0(0.0)	1(100.0)	1(1.2)
*S. aureus*	13(48.1)	14(51.9)	27(31.4)
*E. faecalis*	2(66.7)	1(33.3)	3(3.5)
Total	40(46.5)	46(53.5)	86(100.0)

**Table 3:** multiple antibiotic resistance indices (MARI) of the bacterial uropathogens

Uropathogens	MARI	Antibiotics to which the isolates are resistant
*E. coli*	1.0	NAL, CIP, OFL, LEV, MOX
*K. pneumoniae*	1.0	NAL, CIP, OFL, LEV, MOX
*K. oxytoca*	0.8	NAL, CIP, LEV, MOX
*P. mirabilis*	0.4	LEV, MOX
*P. vulgaris*	0.2	NAL
*S. aureus*	1.0	NAL, CIP, OFL, LEV, MOX
*E. faecalis*	1.0	NAL, CIP, LEV

NAL=nalidixic acid; CIP=ciprofloxacin; OFL=ofloxacin; LEV=levofloxacin; MOX= moxifloxacin

Foot note: FQs=fluoroquinolones; n=number; %=percentage

**Factors associated with fluoroquinolones´ resistant UTI:** when the predictor variables for resistance to fluoroquinolones were subjected to bivariate analysis, they had the following logistic regression values: hospitalization (OR = 4.263; 95% CI: 1.690-10.757; p < 0.05), female gender (OR = 0.279; 95% CI: 0.095-0.817; p < 0.05), indwelling catheter (OR = 4.111; 95% CI: 1.580-10.699; p < 0.05), fluoroquinolones use in the last 12 months (OR = 5.448; 95% CI: 2.153-13.785; p < 0.05), chronic respiratory disease (OR = 5.571; 95% CI: 2.018-15.383; p < 0.05), wrong prescription of antibiotics (OR = 2.636; 95% CI: 1.028-6.758; p < 0.05) and incomplete dose of antibiotics (OR = 3.095; 95% CI: 1.194-8.019; p < 0.05) were found to bear statistically significant relationships (p < 0.05) with fluoroquinolone resistant UTI (Table 4, Table 5).

**Table 4:** bivariate analysis between socio-demographic variables and fluoroquinolone resistant UTI

Variables	Categories	Unadjusted Odds ratio	95% CI	p-value
Department	In-patients	4.263	1.690-10.75	0.002
	Out-patients	1		
Age	≤19 years	1.414	0.433-4.620	0.566
	≥20years	1		
Gender	Female	0.279	0.095-0.817	0.020
	Male	1		
Residence	Rural	0.791	0.306-2.046	0.629
	Sub-urban	4.151	0.821-20.989	0.085
	Urban	1		
Marital status	Married	0.56	0.195-1.617	0.285
	Single	0.234	0.025-2.232	0.207
	Others	1		
Level of education	No education	0.867	0.318-2.360	0.779
	Primary	0.798	0.240-2.649	0.712
	Secondary	2.677	0.650-11.026	0.173
	Tertiary	1		
Circumcision	Yes	0.385	0.020-7.404	0.527
	No	1		
Sexual intercourse	Yes	0.438	0.153-1.251	0.123
	No	1		

Foot note: CI=confidence interval, p=probability, p≤0.05 value is statistically significant under logistic regression

**Table 5:** bivariate analysis between health condition and fluoroquinolone resistant UTI

Variables	Categories	Unadjusted Odds ratio	95% CI	p-value
Pregnancy	Yes	2.121	0.726-6.200	0.169
	No	1		
Hypertension	Yes	0.838	0.264-2.661	0.765
	No	1		
Genitourinary abnormalities	Yes	1.190	0.509-2.782	0.687
	No	1		
Indwelling catheter	Yes	4.111	1.580-10.699	0.004
	No	1		
Diabetes mellitus	Yes	0.838	0.264-2.661	0.765
	No	1		
HIV	Yes	0.619	0.167-2.293	0.473
	No	1		
Fluoroquinolones use in the last 12 months	Yes	5.448	2.153-13.785	0.000
	No	1		
Abortion	Yes	0.179	0.021-1.553	0.119
	No	1		
Chronic respiratory disease	Yes	5.571	2.018-15.383	0.001
	No	1		
Previous surgery	Yes	0.851	0.179-4.055	0.840
	No	1		
Recurrent UTI	Yes	0.560	0.237-1.321	0.186
	No	1		
Previous hospital admission	Yes	0.555	0.198-1.556	0.263
	No	1		
Family history of UTI	Yes	1.176	0.347-3.987	0.794
	No	1		
Previous UTI	Yes	0.778	0.331-1.827	0.564
	No	1		
Wrong prescription	Yes	2.636	1.028-6.758	0.044
	No	1		
Incomplete dose	Yes	3.095	1.194-8.019	0.020
	No	1		

Foot note: CI=confidence interval, p=probability, p≤0.05 value is statistically significant under logistic regression

When the bivariate significant predictor variables for fluoroquinolone resistant UTI were subjected to multiple regression analysis, they had the following logistic regression values: hospitalization (OR = 6.532; 95% CI: 1.653-25.814; p < 0.05), fluoroquinolones use in the last 12 months (OR = 5.349; 95% CI: 1.319-21.699; p < 0.05) and wrong prescription of antibiotics (OR = 4.507; 95% CI: 1.156-17.572; p < 0.05) were found to have statistically significant relationships (p < 0.05) with fluoroquinolone resistant UTI (Table 6). However, age, residence, marital status, level of education, circumcision, sexual intercourse, pregnancy, hypertension, genitourinary abnormalities, diabetes mellitus, HIV, abortion, previous surgery, recurrent UTI, previous hospital admission, family history of UTI and previous UTI were found to have no significant association with fluoroquinolone resistant UTI (Table 4, Table 5).

**Table 6:** factors associated with fluoroquinolone resistant UTI using stepwise forward multiple logistic regression analysis

Variables	Categories	Adjusted Odds ratio	95% CI	p-value
Department	In-patients	6.532	1.653-25.814	0.007
	Out-patients	1		
Fluoroquinolones use in the last 12 months	Yes	5.349	1.319-21.699	0.019
	No	1		
Wrong prescription of antibiotics	Yes	4.507	1.156-17.572	0.030
	No	1		

Foot note: CI=confidence interval, p=probability, p≤0.05 value is statistically significant under logistic regression

## Discussion

This study determined the fluoroquinolone resistance profiles, MARI, factors associated with fluoroquinolone resistance and their strength to the commonly encountered uropathogens among patients attending hospitals in Bushenyi District, Uganda. Data from this study provides an important platform for comparing and monitoring the level of antimicrobial resistance among bacterial uropathogens to guide policy and clinicians on the selection of the most appropriate drugs for managing UTIs. There are reports of increasing antimicrobial resistance globally [30-32]. In spite of the fact that, the tested fluoroquinolones in this study had considerable efficacy, some bacterial uropathogens demonstrated resistance to the fluoroquinolones under study. However, in particular ciprofloxacin and levofloxacin demonstrated high sensitivities. This finding is comparable with other studies done locally and internationally indicating high levofloxacin [22, 33, 34] and high ciprofloxacin [33, 34] sensitivities. Ciprofloxacin exhibited the least resistance of 23/86 (26.7%), a more higher resistance of 40/82 (48.8%) to ciprofloxacin was reported in a study carried out in community acquired uropathogens in Gulu, Northern Uganda [22] and 23/57 (40.4%) in a study carried out to determine bacteriuria among adult non-pregnant women attending Mulago hospital assessment centre in Uganda [23].

While our findings was more comparable to the findings elsewhere: 59/212 (27.8%) [28]. Furthermore, a lesser resistance of 167/1906 (8.8%) to ciprofloxacin by bacterial uropathogens has been reported [33]. Our analysis, importantly demonstrated that, the most resisted fluoroquinolone was ofloxacin with 29/83 (34.9%), this findings is higher than the reports of 345/1906 (18.1%) resistance to ofloxacin [33] and much more similar to 42/155 (27.1%) reported by Prakash and Saxena [35]. The high resistance manifested to ofloxacin could be due to the nature of the study participants such as diabetes, elderly, pregnant women, HIV and infant used in this study which were probably prone to recurrent UTI and subsequent therapeutic usage of this drug previously which might have led to a higher resistance. The resistance of moxifloxacin 27/83 (32.5%) shown in this study is slightly lower than 52/107 (48.6%) reported by Abouelfetouh *et al*. [36]. The highest level of resistance 54/86 (62.8%) shown by the first generation quinolone, nalidixic acid is in conformity with studies done locally and worldwide [4, 22, 23, 33, 37].

As regards the Gram negative bacterial uropathogens, *E. coli* which is known to be the leading bacterial uropathogen [26, 38] emerged the most resistant organism to fluoroquinolones, with the resistance of: 12/36 (33.3%) ciprofloxacin, 16/36 (44.4%) ofloxacin, 11/36 (30.6%) levofloxacin and 13/36 (36.1%) moxifloxacin. Several studies have reported *E. coli* resistance to all or some of these antibiotics [31, 34, 39-41]. Furthermore, among the most frequently encountered bacterial uropathogens, *E. coli* showed the highest frequency of 19/36 (52.8%) fluoroquinolones´ resistant strains. This particular finding is in conformity with other studies [11, 15]. All the other bacterial uropathogens with *E. coli* inclusive have shown high level of resistance to nalidixic acid. This finding is supported by several other studies done locally and internationally [4, 22, 23, 33, 35, 37]. To halt the progress of resistance to fluoroquinolones, antibiotic susceptibility to the first generation quinolones such as nalidixic should be considered. All the bacterial uropathogens tested against the fluoroquinolones showed MARI of ≥ 0.2. Multiple antibiotic resistance index is a tool used to assess the spread of bacterial resistance in a specified population [42, 43].

Values of MARI ≥0.2 indicates that bacterial strains of this nature originate from an ecological system where multiple antibiotics are being used or abused [44-46]. This is a clear indication that a huge proportion of these bacterial uropathogens were exposed to multiple antibiotics and this, resulted into development of resistance to these drugs. Similar incidences of resistance, though to different sets of antibiotics have been reported elsewhere [28,44]. Furthermore, reports of resistance of bacterial uropathogens to commonly used antibiotics have been documented [35]. This study demonstrated that fluoroquinolone use in the last 12 months, hospitalization, female gender, indwelling catheter, chronic respiratory disease, wrong prescription and incomplete dose of antibiotics were found to bear statistically significant relationships (p < 0.05) with fluoroquinolone resistant UTI. This study demonstrated that prior use of fluoroquinolones in the last 12 months bears statistically significant relationship (OR=5.448; 95% CI: 2.153-13.785; p < 0.05) with development of fluoroquinolone resistant UTI. Our findings are comparable with other previous reports [47-52].

Our study also found, a statistical significant relationship (OR=4.111; 95% CI: 1.580-10.699; p < 0.05) between fluoroquinolone resistant UTI and presence of indwelling catheter, this is in agreement with other studies [48-50]. This study also, demonstrated a significant statistical relation (OR=3.095; 95% CI: 1.194-8.019; p < 0.05) between incomplete dose of antibiotics and fluoroquinolone resistant UTI. These findings are in conformity with a report of development of resistance to antibiotics by Bhattacharjee [53]. In infection management, it´s much easier to treat low level antimicrobial resistance because the pathogen can be eliminated by the antimicrobial agent of the usual dose. However, in an event of incomplete dose of antibiotic administration, selection of low level bacterial resistance can occur much easily, that can later on translate into new mutations and eventually resulting into gradual selection and emergence of incredibly high level of resistance to antibiotics that will not respond to treatment by the usual therapeutic dose of that particular antibiotic which was sufficient before [53].

Additionally, our study also found out that wrong prescription of antibiotics bear statistically significant relationship (OR = 2.636; 95% CI: 1.028-6.758; p < 0.05) with fluoroquinolone resistant UTI, this could be due to the high used of over the counter antibiotics, bought from unlicensed drug stores and in open markets and self-medication in Uganda [54]. This study also found, a statistical significant association (OR = 0.279; 95% CI: 0.095-0.817; p < 0.05) between female gender and fluoroquinolone resistant UTI. However in contrast, this finding deviated from other studies done elsewhere [4, 55]. Furthermore, our study demonstrated that hospitalization had a statistical significant association (OR=4.263; 95% CI: 1.690-10.757; p < 0.05) with fluoroquinolone resistant UTI. This finding is supported by a study done previously by Nicoletti *et al*. [52]. Finally in our study, chronic respiratory disease was found to have a statistical significant relationship (OR=5.571; 95% CI: 2.018-15.383; p<0.05) with fluoroquinolone resistant UTI. This finding is comparable with studies done elsewhere [56-59]. Our study had the following limitations: insufficient data on the previous patients´ antimicrobial use as some of them did not have records of previous medications. Also, we did not distinguish, recurrent, uncomplicated and complicated UTIs. Therefore, the resistance pattern in these subjects couldn´t be attained.

## Conclusion

Ofloxacin and moxifloxacin were the most resisted fluoroquinolones after nalidixic acid. The considerable increase in the values of MARI, underscores the urgent need for continuous local surveillance and antibiotic susceptibility testing of the common bacterial uropathogens implicated in UTIs before antibiotic prescription by clinicians. Also, multiple logistic regression revealed that hospitalization, history of fluoroquinolones use in the last 12 months and wrong prescription of antibiotics were found to bear statistically significant relationships (p < 0.05) with fluoroquinolone resistant UTI. To halt the progress of bacterial uropathogens´ resistance to fluoroquinolones, we recommend routine antibiotic susceptibility testing to the first generation quinolones such as nalidixic acid to avoid fluoroquinolone abuse in hospitalized patients, patients with history of fluoroquinolones use in the last 12 months and wrong prescription of antibiotics. For empiric treatment of UTIs in Bushenyi District, ciprofloxacin still remains the first line of choice among the fluoroquinolone class of antibiotics.

### What is known about this topic

- Prevalence of the bacterial uropathogens among patients attending hospitals in Bushenyi District, Uganda;
- *Escherichia coli* was the most predominant bacterial uropathogens isolated from patients attending hospitals in Bushenyi District, Uganda;
- Factors associated with UTIs among patients attending hospitals in Bushenyi District, Uganda.

### What this study adds

- Bacterial uropathogens´ fluoroquinolone resistant profiles among patients attending hospitals in Bushenyi District, Uganda;
- Multiple antibiotic resistance indices of the bacterial uropathogens studied;
- Factors associated with fluoroquinolone resistant UTIs among patients attending hospitals in Bushenyi District, Uganda.
